# Mechanisms of Long Non-Coding RNAs in Cancers and Their Dynamic Regulations

**DOI:** 10.3390/cancers12051245

**Published:** 2020-05-15

**Authors:** Xiao-Zhen Zhang, Hao Liu, Su-Ren Chen

**Affiliations:** Education Key Laboratory of Cell Proliferation & Regulation Biology, College of Life Sciences, Beijing Normal University, Beijing 100875, China; zhangxz91@yeah.net (X.-Z.Z.); liuhaobnu89@yeah.net (H.L.)

**Keywords:** lncRNA, cancer, mechanism, turnover, secondary structure, modification, autophagy, review

## Abstract

Long non-coding RNA (lncRNA), which is a kind of noncoding RNA, is generally characterized as being more than 200 nucleotide transcripts in length. LncRNAs exhibit many biological activities, including, but not limited to, cancer development. In this review, a search of the PubMed database was performed to identify relevant studies published in English. The term “lncRNA or long non-coding RNA” was combined with a range of search terms related to the core focus of the review: mechanism, structure, regulation, and cancer. The eligibility of the retrieved studies was mainly based on the abstract. The decision as to whether or not the study was included in this review was made after a careful assessment of its content. The reference lists were also checked to identify any other study that could be relevant to this review. We first summarized the molecular mechanisms of lncRNAs in tumorigenesis, including competing endogenous RNA (ceRNA) mechanisms, epigenetic regulation, decoy and scaffold mechanisms, mRNA and protein stability regulation, transcriptional and translational regulation, miRNA processing regulation, and the architectural role of lncRNAs, which will help a broad audience better understand how lncRNAs work in cancer. Second, we introduced recent studies to elucidate the structure of lncRNAs, as there is a link between lncRNA structure and function and visualizing the architectural domains of lncRNAs is vital to understanding their function. Third, we explored emerging evidence for regulators of lncRNA expression, lncRNA turnover, and lncRNA modifications (including 5-methylcytidine, N6-methyladenosine, and adenosine to inosine editing), highlighting the dynamics of lncRNAs. Finally, we used autophagy in cancer as an example to interpret the diverse mechanisms of lncRNAs and introduced clinical trials of lncRNA-based cancer therapies.

## 1. Introduction

The central dogma of the molecular biology of DNA-RNA-protein proposes that genetic information is stored in protein-coding genes [1]. From the traditional point of view, proteins are the main performers of cellular function, while RNA is considered to be an intermediary between genes and proteins. Surprisingly, the international Human Genome Project (HGP) has revealed that more than 90% of the genome is comprised of non-coding genes [2,3].

Recent transcriptome sequencing has discovered, across various species, thousands of long non-coding RNAs (lncRNAs), which are most commonly defined as non-protein-coding RNA molecules longer than 200 nucleotides. LncRNAs can be categorized as intergenic, intronic, sense, or antisense, according to their location and transcription pattern. Intergenic lncRNAs refers to lncRNAs that are transcribed from regions between protein-coding genes, whereas those transcribed from the introns of genes are known as intronic lncRNAs. Transcribed lncRNAs from the sense strand of protein-coding genes are termed sense lncRNAs, and antisense lncRNAs, on the contrary, are transcribed from the antisense strand [4,5].

**Figure 1:**
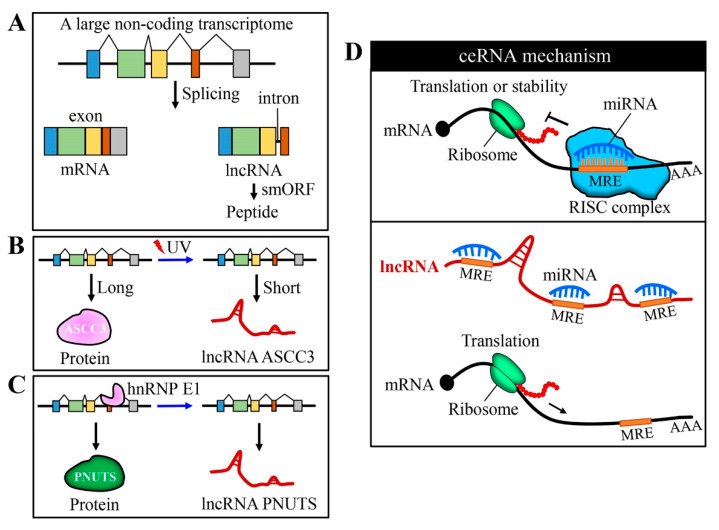
Generation of long non-coding RNAs (lncRNAs) and their function as competing endogenous RNA (ceRNA). (**A**) Alternative splicing gives rise to the complex mammalian genome; lncRNA variants may arise from the skipping of the first or last exon, resulting in the loss of start or stop codons. A part of lncRNAs harbors small open reading frames (smORF) and can encode short peptides with a function. (**B**) Protein and lncRNA can sometimes be encoded by the same gene. Exposure to UV light leads to a slowdown of transcript elongation and the *ASCC3* gene expresses a shorter RNA isoform (lncRNA ASCC3), instead of protein. (**C**) The binding of heterogeneous nuclear ribonucleoprotein E1 (hnRNPE1) to a nucleic element within exon 12 of the serine/threonine-protein phosphatase 1 regulatory subunit 10 (*PNUTS*) gene that regulates its alternative splicing to generate the lncRNA PNUTS. (**D**) In general, miRNAs bind to the 3′ untranslated (UTR) region of target mRNAs and regulate their expression at a post-transcription level (inhibiting translation and/or decreasing the stability of mRNA). LncRNAs can act as sponges for miRNA through their binding sites and inhibit the function of miRNA on its target gene(s).

Coding and non-coding genes are generally classified by their protein-coding potential or whether the transcripts need to be translated to protein to perform their functions. Both a large non-coding transcriptome and extensive alternative splicing contribute to the complex genome and generation of a great diversity of lncRNAs [6,7]. Interestingly, recent studies have identified lncRNAs that harbor small open reading frames to encode short functional peptides [8,9,10] (Figure 1A). Protein and lncRNA can be encoded by the same gene. For example, exposure to ultraviolet (UV) light leads to a slowdown of transcript elongation; upon UV irradiation, the activating signal co-integrator complex 3 (*ASCC3*) gene expresses a shorter RNA isoform (lncRNA ASCC3) that mediates transcription recovery after UV-induced DNA damage [11] (Figure 1B). Furthermore, Grelet et al. characterized the binding of heterogeneous nuclear ribonucleoprotein E1 (hnRNPE1) to the DNA element in exon 12 of the serine/threonine-protein phosphatase 1 regulatory subunit 10 (*PNUTS*) gene that regulates its alternative splicing to generate lncRNA PNUTS [12] (Figure 1C).

Until now, many cancer risk-associated loci have been explored in large-scale genome-wide association studies (GWASs). Notably, a high proportion of GWAS-identified cancer susceptibility gene variants are located in the non-coding regions of the genome [13,14,15,16,17,18]. LncRNAs, such as RP11-403A21.1 at 18q11.2, are highly associated with the human ovarian cancer risk [19]. Forty-five candidate lncRNAs have been identified in GWASs as being associated with the risk for prostate cancer [20]. Cyclin D1 (CCND1)-upstream intergenic DNA repair 1 and 2 (lncRNA CUPID1/2) mediates the breast cancer risk at 11q13 by modulating the cellular response to DNA damage [21]. The role of lncRNAs in cancer biology is continually expanding; importantly, some cancer-associated lncRNAs are clinically relevant. For example, a recent study identified 1145 temporally expressed S-phase-enriched lncRNAs, and silencing of some of them in cancer models affects crucial cancer cell hallmarks [22]. Moreover, a small subset of lncRNAs has been identified as being strongly correlated with the treatment response and survival in acute myeloid leukemia [23]. Bromodomain and extra-terminal motif (BET) inhibitors have been demonstrated to be a promising new therapy in several cancer types, and the lncRNA pharmacogenomics landscape suggests that epigenetically-induced lncRNA1 (EPIC1) stimulates BET inhibitor resistance via the activation of MYC proto-oncogene, bHLH transcription factor (MYC) transcription [24]. Collectively, lncRNAs have emerged as novel regulators of human cancer development; however, the mechanism by which lncRNA exerts its molecular function remains largely uncharacterized.

In this up-to-date review, we mainly summarize the diverse mechanisms of lncRNA actions in cancer pathology and discuss the turnover, secondary structure, and modifications of lncRNA. Finally, we briefly introduce the lncRNAs involved in autophagy in cancer and clinical trials of lncRNA-based cancer therapies. We hope that the current review, summarizing the regulatory mechanisms of lncRNAs and their dynamic regulations in cancer biology, will be of a certain significance for researchers, doctors, and graduate students in the field of lncRNA and cancer, and allow the general public to obtain useful and timely information. We need to point out that many interesting topics relating to lncRNAs are not included here due to the focus of this review, such as lncRNAs and epithelial–mesenchymal transition (EMT), lncRNAs and cancer cell stemness, lncRNAs and chemo/radioresistance, clinical trials involving lncRNAs, lncRNA delivery strategies, lncRNAs as biomarkers, exosomal lncRNAs and cell–cell communication, and lncRNA database collection.

## 2. Regulatory Mechanisms of LncRNAs in Cancer Progression

### 2.1. Competing Endogenous RNA (ceRNA) Mechanism

MiRNAs bind to sequences with partial complementarity on target RNA transcripts using miRNA recognition elements (MREs). LncRNAs can act as sponges for miRNA through their MREs and modulate the activity of miRNA on its target mRNAs. LncRNAs compete with mRNA targets for binding to miRNAs, thus relieving the inhibitory action of miRNAs on mRNA targets [25,26] (Figure 1D). Recent studies have characterized dysregulated lncRNA-miRNA-mRNA networks based on ceRNA theory. We briefly summarized recent studies adopting the ceRNA mechanism to explain the action of lncRNA (Table 1).

PTEN pseudogene (lncRNA PTENP1) is derived from its homologous protein-coding gene phosphatase and tensin homolog (*PTEN*) due to a missense mutation of the methionine (Met) codon preventing translation. LncRNA PTENP1 acts as a decoy for PTEN-targeting miRNAs to regulate *PTEN* expression [27]. LINC00673 sponges miR-150-5p to modulate the expression of zinc finger E-box binding homeobox 1 (ZEB1), a key epithelial–mesenchymal transition regulator, in non-small cell lung cancer [28]. Osteosarcoma doxorubicin-resistance related up-regulated lncRNA (ODRUL) combines with miR-3182 to elevate matrix metalloproteinase 2 (MMP2) expression in osteosarcoma [29]. In gastric cancer, LINC01234 functions as a ceRNA to regulate core-binding factor β (CBFB) expression by sponging miR-204-5p [30,31]. Colorectal cancer progression is promoted by nuclear paraspeckle assembly transcript 1 (lncRNA NEAT1), which competitively binds miR-34a with histone deacetylase sirtuin 1 (SIRT1) [32]. In ovarian cancer, the WD repeat and FYVE domain containing 3-antisense 2 (lncRNA WDFY3-AS2) sponges miR-18a to upregulate nuclear receptor RAR-related orphan receptor A (RORA) [33].

Importantly, there are several issues that should be noted: (i) whether lncRNAs effectively function as ceRNAs at physiologically relevant copy numbers is debatable because most of the studies describing miRNA sponges rely on overexpression; (ii) whether ceRNA theory is truly the principle mechanism of lncRNA function is sometimes questionable, and the reason why a large amount of literature claims ceRNAs as the mechanism of lncRNA action; (iii) whether the loss of lncRNAs is precisely responsible for the phenotype needs to be confirmed because some lncRNAs harbor small open reading frames and encode functional short peptides [10,34,35,36,37]; (iv) the ceRNA effect is affected by the abundance of miRNAs and lncRNAs, the number and affinity of binding sites, and the degree of miRNA/mRNA complementarity [38,39]. Further studies are therefore needed to determine the molecular mechanisms by which miRNA will be sponged or activated to degrade its target transcripts.

### 2.2. Epigenetic Regulation by lncRNAs

In addition to the ceRNA mechanism, lncRNAs directly participate in cancer epigenetic regulation via interacting with key histone-modification enzymes [40]. Current studies have identified that lncRNAs can modulate downstream gene expression through interacting with chromatin remodeling complexes, including polycomb repressive complexes (PRCs), mixed-lineage leukemia 1 (MLL1), and SWI/SNF complexes.

PRCs are composed of PRC1 and PRC2 and modify histones to mediate gene silencing [41,42]. The PRC1 complex mainly contains Bmi1/Mel18, mPh1/2, Pc/Chromobox (CBX), and the ubiquitin E3 ligase RING1A/B, whereas the PRC2 complex is formed of EED, Suz12, and methyltransferase EZH2. Notably, a large proportion of lncRNAs (~20%) expressed in various cell types are found in association with the PRC2 complex [43]. More than 9000 lncRNAs associated with PRC2 in embryonic stem cells have been identified by RNA binding protein immunoprecipitation (RIP)-seq analysis [44]. Here, we briefly summarize recent studies on the epigenetic regulation conducted by lncRNAs in cancer progression (Table 2).

**Figure 2:**
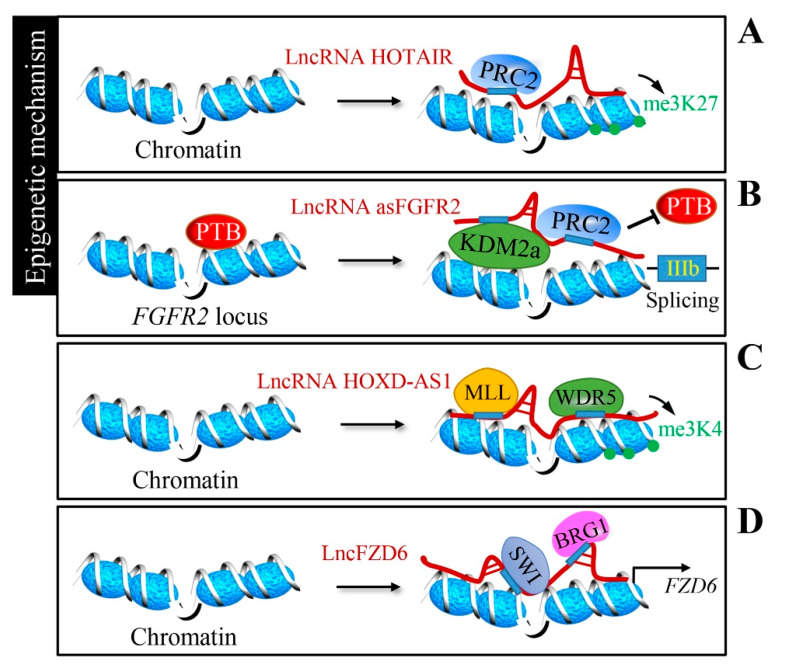
Epigenetic regulation by lncRNAs. (**A**) LncRNA HOX transcript antisense intergenic RNA (HOTAIR) regulates *HOXD* cluster genes via the recruitment of the polycomb repressive complex 2 (PRC2) silencing complex to execute gene silencing by histone H3 lysine 27 tri-methylation (H3K27me3). (**B**) LncRNA FGFR2-AS recruits the PRC2 complex and histone demethylase KDM2a to the *FGFR2* locus, generating a splicing-specific chromatin signature. (**C**) LncRNA HOXD antisense growth-associated long non-coding RNA (HOXD-AS1) recruits the WD repeat domain 5 (WDR5)-mixed-lineage leukemia (MLL) complex and mediates histone H3 lysine 4 tri-methylation (H3K4me3) to directly regulate the expression of target genes. (**D**) Divergent lncRNA of FZD6 (LncFZD6) recruits the BRG1-embedded SWI/SNF complex to the *FZD6* promoter and drives *FZD6* transcription.

Antisense non-coding RNA in the INK4 locus (ANRIL) binds to CBX7 within the PRC1 complex to form heterochromatin surrounding the *INK4b-ARF-INK4a* locus, leading to its repression in prostate cancer [45]. HOX transcript antisense intergenic RNA (HOTAIR) is well-known lncRNA located in the *HOXC* gene cluster and regulates *HOXD* cluster genes on a different chromosome via the recruitment of the PRC2 complex with its 5′-end to mediate transcriptional silencing [46] (Figure 2A). LncRNA plasmacytoma variant translocation 1 (PVT1) promotes gastric cancer cell proliferation by targeting the occupancy of EZH2 within the PRC2 complex to epigenetically regulate *p15* and *p16* [47]. A conserved sequence element in the long intergenic non-protein-coding RNA p53-induced transcript (LINC-PINT) has been shown to mediate LINC-PINT-PRC2 interaction, which is necessary for the repression of pro-invasive genes [48]. Furthermore, lncRNAs can modulate chromatin signatures to establish alternative splicing; fibroblast growth factor receptor 2 (FGFR2)-antisense (LncRNA FGFR2-AS) generates a splicing-specific chormatin signature to favor exon IIIb inclusion in epithelial cells through recruiting PRC2 and histone demethylase KDM2a to the *FGFR2* locus [49,50] (Figure 2B).

MLL1 is a histone H3Lys4 (H3K4) methyltransferase and forms a complex with WD repeat domain 5 (WDR5) and other components. WDR5 has been proven to associate with lncRNAs to preserve the activation of chromatin [59,60]. HOXD antisense growth-associated long non-coding RNA (HOXD-AS1) promotes the proliferation and chemoresistance of prostate cancer by recruiting WDR5 to regulate target genes via H3K4 tri-methylation [51] (Figure 2C). Gastric cancer-associated WDR5 and KAT2A binding lncRNA (GCAWKR) modulates the affinity for WDR5/KAT2A complexes in the promoter region of target genes [52]. In hepatocarcinogenesis, enhanced growth arrest specific 8 (*GAS8*) transcription is achieved by recruitment of the MLL1/WDR5 complex via lncRNA GAS8-AS1 [53]. The SWI/SNF complex is another well-studied chromatin remodeling complex. Divergent lncRNA of FZD6 (lncFZD6) recruits the BRG1 (Brahma-related gene 1; SMARCA4)-embedded SWI/SNF complex to the *FZD6* promoter, driving the *FZD6* transcription in liver cancer [54] (Figure 2D).

Moreover, lncRNAs serve as a scaffold to recruit other chromatin-remodeling complexes or histone acetyl/methyl-transferases to target chromatin, including E1A binding protein p300 (p300), the INO80 complex ATPase subunit (INO80), DNA methyltransferase 1 (DNMT1), euchromatic histone lysine methyltransferase 2 (G9a), and the *Drosophila* nucleosome-remodeling factor (NURF). An antisense transcript of SATB homeobox 2 (SATB2) (lncRNA SATB2-AS1) could serve as a scaffold to recruit histone acetyltransferase p300 to downregulate SATB2 expression and promote colorectal carcinoma progression [55]. Liver cancer stem cell self-renewal requires the recruitment of chromatin-remodeling complex INO80 to the *BMPR1A* promoter by the action of an antisense transcript of HAND2 (lncRNA HAND2-AS1) [56]. Similarly, liver tumor-initiating cell maintenance requires HOXA10 transcription by the lncHOXA10-mediated recruitment of chromatin remodeling complex NURF [57]. The antisense transcript of PYD and CARD domain containing (PYCARD) (lncRNA PYCARD-AS1) could recruit DNMT1 and histone methyltransferase G9a to the *PYCARD* promoter to further regulate apoptosis [58].

### 2.3. Decoy Mechanism

**Figure 3:**
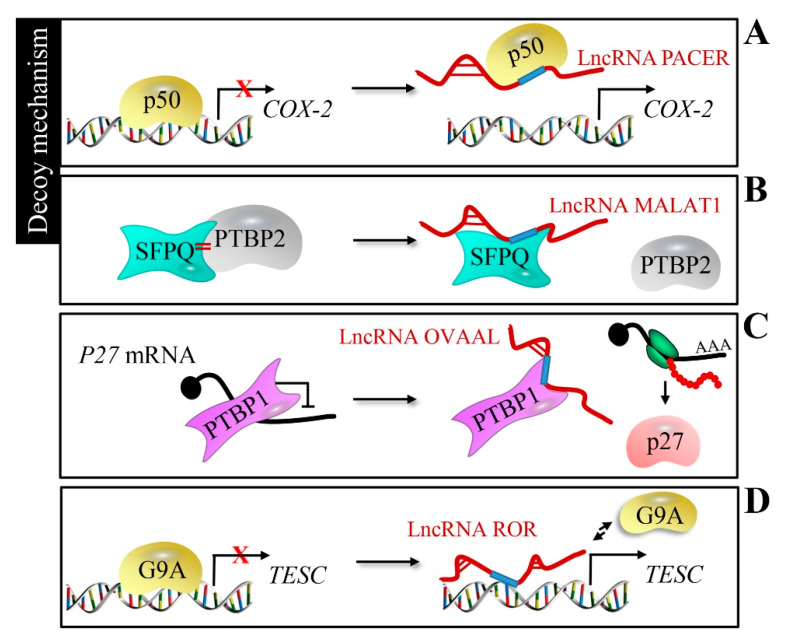
Decoy mechanism of lncRNAs. (**A**) LncRNA PACER interacts with the p50 to separate it from the cytochrome c oxidase subunit II (*COX-2*) promoter, activating *COX-2* gene expression. (**B**) LncRNA MALAT1 binds to SFPQ and releases oncogene PTBP2 from the SFPQ/PTBP2 complex. (**C**) LncRNA ovarian adenocarcinoma-amplified lncRNA (OVAAL) with *p27* mRNA competitively binds to PTBP1 to release the inhibition of *p27* expression. (**D**) LncRNA ROR competes with histone G9A methyltransferase at the tescalcin (*TESC*) promoter, and free G9A fails to methylate the *TESC* promoter.

LncRNAs can function as a decoy of transcription factors to sequester from their DNA-binding sequences, in order to regulate target gene expression. For example, P50-associated COX-2 extragenic RNA (lncRNA PACER) separates the repressive nuclear factor kappa B subunit 1 (NF-kB) subunit p50 from the cytochrome c oxidase subunit II (*COX-2*) promoter to activate *COX-2* gene expression [61] (Figure 3A). Colorectal cancer growth and metastasis could be promoted by the release of oncogene PTBP2 from the SFPQ/PTBP2 complex, which is achieved by the affinity between human metastasis-associated lung adenocarcinoma transcript 1 (lncRNA MALAT1) and SFPQ [62] (Figure 3B). Ovarian adenocarcinoma-amplified lncRNA (OVAAL) binds to polypyrimidine tract-binding protein 1 (PTBP1) and reduces the recruitment of PTBP1 to the internal ribosome entry site of *p27* mRNA, thus imparting an inhibitory effect on *p27* mRNA translation in cancer cells [63] (Figure 3C). LncRNAs can also repel endogenous histone methyltransferase; for example, the regulator of reprogramming (lncRNA ROR) competes with histone G9A methyltransferase at the tescalcin (*TESC*) promoter, inducing aberrant TESC expression to trigger gastric and colon tumorigenesis [64] (Figure 3D).

### 2.4. LncRNA Control of mRNA and Protein Stability

**Figure 4:**
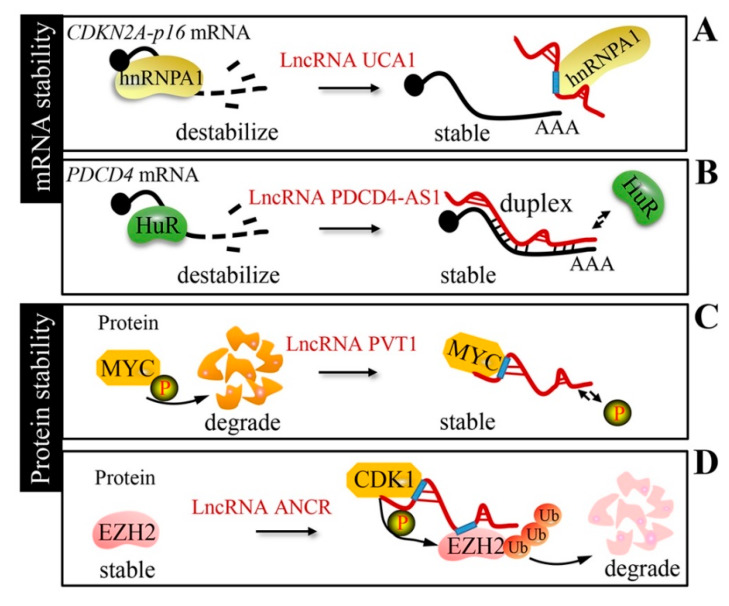
Stability mechanism. (**A**) LncRNA UCA1 stabilizes *CDKN2A-p16* mRNA by sequestering heterogeneous nuclear ribonucleoprotein A1 (hnRNPA1). (**B**) LncRNA PDCD4-AS1 forms an RNA duplex with *PDCD4* mRNA to expel ELAV-like RNA binding protein 1 (HuR). (**C**) LncRNA plasmacytoma variant translocation 1 (PVT1) protects the MYC protein from phosphorylation-mediated degradation. (**D**) LncRNA ANCR interacts with EZH2 and promotes the CDK1-mediated phosphorylation and degradation of EZH2.

Although proteins are the main executors of cellular function, nearly half of the changes in expression occur at the RNA level, specifically through the control of RNA stability [65]. Accumulating evidence suggests that lncRNAs are novel regulators of the mRNA stability of oncogenes or cancer suppressor genes (Table 3). Kumar et al. indicated that urothelial carcinoma associated 1 (lncRNA UCA1) stabilizes *CDKN2A-p16* mRNA by sequestering heterogeneous nuclear ribonucleoprotein A1 (hnRNPA1) [66] (Figure 4A). ELAV-like RNA binding protein 1 (HuR), an RNA-binding posttranscriptional regulator, affects mRNA stability through binding to the RNA motif and is implicated in cancer formation, progression, and metastasis. Programmed cell death 4 (PDCD4)-antisense RNA1 (lncRNA PDCD4-AS1) stabilizes *PDCD4* mRNA by forming an RNA duplex and increases the interaction between *PDCD4* mRNA and HuR in breast cancer [67] (Figure 4B).

In addition to being an mRNA stability regulator, lncRNAs have been reported to protect protein stability (Table 3). LncRNA PVT1 has no effect on *MYC* mRNA stability, but protects the MYC protein from phosphorylation-mediated degradation in 8q24-amplified human cancer cells [68] (Figure 4C). In triple-negative breast cancer, long intergenic non-coding RNA for kinase activation (LINK-A) mediates hypoxia inducible factor 1 subunit alpha (HIF1α) phosphorylation and stabilization by protein tyrosine kinase 6 (BRK) and leucine rich repeat kinase 2 (LRRK2) interaction [69]. On the other hand, lncRNAs can interact with proteins and limit protein stability via degradation in cancers. Anti-differentiation non-coding RNA (lncRNA ANCR) suppresses breast cancer progression by promoting the cyclin dependent kinase 1 (CDK1)-mediated phosphorylation and degradation of EZH2 [70] (Figure 4D). Growth arrest-specific transcript 5 (lncRNA GAS5) promotes Yes1-associated transcriptional regulator (YAP) degradation via the ubiquitin-proteasome pathway by interacting with the WW domain of the YAP protein in colorectal cancer [71].

### 2.5. Transcriptional and Translational Regulation by LncRNAs

**Figure 5:**
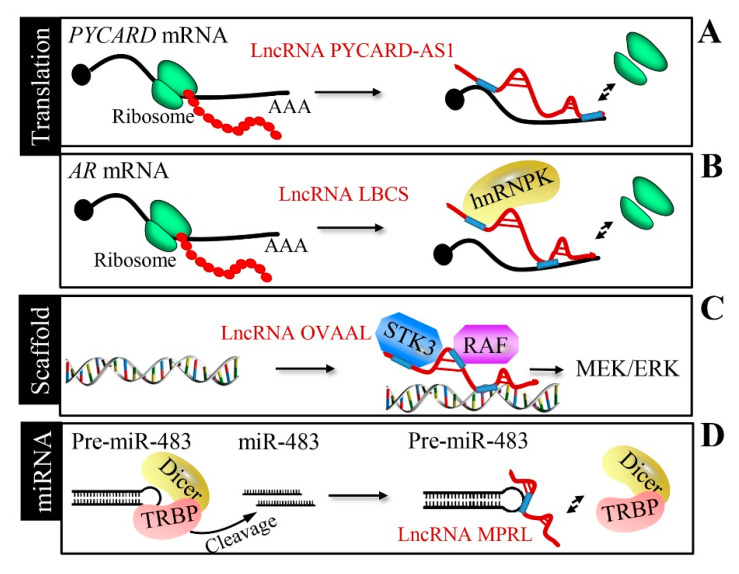
Translation regulation, transcription factor scaffold, and miRNA processing by lncRNAs. (**A**) LncRNA PYCARD-AS1 interacts with *PYCARD* mRNA via the 5′ overlapping region, inhibiting ribosome assembly for *PYCARD* translation. (**B**) LncRNA LBCS suppresses the *AR* translation efficiency by forming a complex with hnRNPK and *AR* mRNA. (**C**) Like a scaffold, lncRNA OVAAL enhances the binding of serine/threonine-protein kinase 3 (STK3) and RAF to trigger MEK/ERK activation. (**D**) LncRNA miRNA processing-related lncRNA (MPRL) inhibits miR-483-5p generation by binding with pre-miR-483 to block pre-miR-483 cleavage by the human immunodeficiency virus transactivating response RNA-binding protein (TRBP)-Dicer complex.

In addition to controlling mRNA stability, the interaction between lncRNA and mRNA can disturb mRNA translation. Antisense to the pro-apoptotic gene PYCARD (lncRNA PYCARD-AS1) inhibits the ribosome assembly for PYD and CARD domain-containing (*PYCARD*) translation by interacting with *PYCARD* mRNA [58] (Figure 5A). A low expression in bladder cancer stem cells (lncRNA LBCS) forms a complex with the RNA-binding protein hnRNPK and androgen receptor (*AR*) mRNA to suppress *AR* translation in prostate cancer [72] (Figure 5B). In addition to the regulation of translation, interestingly, lncRNAs can affect transcription by modulating the local chromatin structure. An antisense lncRNA named BGas interacts with high mobility group proteins HMGA1 and HMGB1, as well as the partner of Y14 and mago (WIBG), to regulate *CFTR* gene transcription through the modulation of its DNA architecture and local chromatin [73].

### 2.6. Scaffold of Transcription Factors by LncRNAs

LncRNA OVAAL physically interacts with serine/threonine-protein kinase 3 (STK3), enhancing the binding between STK3 and the Raf-1 proto-oncogene, serine/threonine kinase (RAF1), in multiple cancers [63] (Figure 5C). The RBM5 anti-sense transcript (lncRNA RBM5-AS1) sustains the function of colon cancer-initiating cells through the organization of a transcriptional complex containing β-catenin and transcription factor 4 (TCF4) [74]. LINC00844 facilitates AR binding to the chromatin to determine prostate cancer progression [75].

### 2.7. miRNA Processing Mechanism

Some lncRNAs, such as mitochondrial dynamic-related lncRNA (MDRL) and miRNA processing-related lncRNA (MPRL), can exert their function by regulating miRNA processing. Nucleus miR-361 disturbs the drosha ribonuclease III (Drosha)-mediated cleavage of pri-miR-484 into pre-miR-484; interestingly, lncRNA MDRL regulates miR-484 processing by antagonizing miR-361 [76]. LncRNA MPRL inhibits the Dicer (dicer 1, ribonuclease III)-mediated processing of miR-483 through affinity interaction with pre-miR-482 [77] (Figure 5D).

### 2.8. Architectural Role of LncRNAs

Recent studies have identified nuclear-enriched abundant transcript 1 (lncRNA NEAT1)-containing structures that are referred to as “paraspeckles”. Paraspeckles are localized in the interchromatin space of cellular nuclei and perform critical functions, including, but not limited to, gene expression network regulation [78,79,80]. Here, we introduce the most recent advances in the understanding of paraspeckle functions. Hypoxia stimulates tumor development, partially through the HIF-2α-mediated transcriptional activation of lncRNA NEAT1 and the formation of nuclear paraspeckles [81]. Furthermore, lncRNA NEAT1 is a promising target for increasing the sensitivity of cancer cells to chemotherapy [82]. LncRNA NEAT1-containing paraspeckles also stimulate interleukin 6 (IL-6)-mediated hepatocellular carcinoma development by the nuclear entrapping of IL-6/signal transducer and activator of transcription 3 (STAT3) inhibitors [83]. A recent study identified the relationship between mitochondrial stress, lncRNA NEAT1 paraspeckles, and mitochondrial homeostasis; lncRNA NEAT1 depletion alters the sequestration of mitochondrial mRNAs in paraspeckles to influence mitochondrial homeostasis [84]. Although lncRNA NEAT1 plays important roles in paraspeckles, the function and regulatory mechanisms of paraspeckles, as well as the involvement of other lncRNAs in biological paraspeckle activities, are largely unknown.

### 2.9. Other Mechanisms of LncRNA Function

LncRNAs can work through other uncommon mechanisms, including, but not limited to, promoter completion, chromatin enrichment, alternative splicing, and 3′ UTR binding. Cho et al. provided interesting data indicating that the lncRNA PVT1 promoter inhibits oncogene MYC expression at the same chromosome via promoter competition [85]. Hemin-induced cheRNA downstream of fetal hemoglobin (lncRNA HIDALGO), a chromatin-enriched lncRNA, tightly associates with chromatin and forms a bridge between the enhancer of HIDALGO transcription start sites and the hemoglobin subunit gamma 1 (*HBG1*) promoter [86]. Gordon et al. have suggested that lncRNA MALAT1 promotes ovarian cancer progression by regulating the splicing factor RNA binding fox-1 homolog 2 (RBFOX2)-mediated alternative splicing of the kinesin family member 1B (*KIF1Bβ*) gene [87]. LncRNAs may function in a manner more analogous to miRNAs; prostate-cancer-associated ncRNA transcript 1 (lncRNA PCAT-1) attenuates the expression of BRCA2 DNA repair associated (BRCA2) via the post-transcriptional repression of *BRCA2* 3′ UTR [88].

## 3. Secondary Structure of LncRNAs

There is a link between lncRNA structure and function; accordingly, visualizing the architectural domains of lncRNAs is vital to understanding their function. One of the best studied lncRNAs is HOX transcript antisense intergenic RNA (HOTAIR), which has been demonstrated to repress tumor and metastasis suppressor genes. Somarowthu et al. [89] purified lncRNA HOTAIR in a homogeneous and monodispersed form to further determine its experimental secondary structure. LncRNA HOTAIR was found to be highly structured and composed of four independent architectural modules, two of which mediate the interaction of lncRNA HOTAIR with PRC2 and LSD1 (RE1-silencing transcription factor). The X-inactive specific transcript (XIST) (∼18 kb) represents one of the earliest discoveries of mammalian lncRNAs, with a well-defined role in the regulation of gene expression and X-chromosome inactivation. The conformation of XIST has been explored by targeted structure-seq in mammalian cells [90]. The repeat A region of XIST is a functional element important for gene repression, whereas the repeat C region mediates the interaction between XIST and proteins that anchor XIST to chromatin. The polyadenylated nuclear (PAN) lncRNA of Kaposi’s sarcoma-associated herpes virus (KSHV) is produced during lytic infection and interacts with a variety of transcriptional regulators and chromatin modifiers. The structure of PAN lncRNA has been resolved and its binding motifs of LANA and ORF57 have been mapped [91]. PAN lncRNA folds into three branched domains and its 5′ Mta responsive element (MRE) region and 3′ expression and nuclear retention element (ENE) are identified in a larger structural context. A recent study identified the secondary structure of MALAT1, an important cancer-related lncRNA, in vitro and in various human cell lines [92]. In addition to providing protein/RNA-binding structural information, this study reveals that the structure of lncRNA MALAT1 can be perturbed by RNA modifications, mutations in cancer, and single-nucleotide polymorphisms. Moreover, recent studies have produced the three-dimensional structure of lncRNAs RepA and 7SK [93,94,95]. Collectively, a major feature of lncRNAs is their propensity to assume higher order structures that mediate diverse functions. The structural map of lncRNAs would help guide experiments and accelerate research that is focused on the molecular functions of lncRNA.

## 4. Dynamic Regulation of LncRNAs in Cancer Biology

### 4.1. Regulators of LncRNA Expression

Recent studies have provided new insights into how lncRNA expression is regulated by various extra- and intra-cellular factors, including growth factors, hormones, DNA damage stress, transcription factors, and histone modifications (Table 4). In breast cancer, the epidermal growth factor (EGF) rapidly attenuates the expression of inhibiting metastasis (lncRNA LIMT) by enhancing histone deacetylation at its promoter region [96]. Growth factor deprivation or terminal differentiation upregulates lncRNAs, termed quiescence-induced lncRNAs, which play important roles in H4K20me3-mediated chromatin compaction and transcriptional silencing [97]. Furthermore, hormone-regulated lncRNAs have been reported to be closely associated with hormone-dependent cancers. An androgen-responsive C-terminal binding protein 1 antisense (lncRNA CTBP1-AS) recruits the PTB-associated splicing factor (PSF) together with histone deacetylase (HDAC)/ SIN3 transcription regulator family member A (Sin3A ) to the promoter of androgen receptor corepressor C-terminal binding protein 1 (*CTBP1*) and attenuates its expression in prostate cancer [98]. During the progression of renal cell carcinoma, estrogen receptor β regulates lncRNA HOTAIR expression, further antagonizing the miR138/200c/204/217 function [99]. Niknafs et al. observed that Down syndrome cell adhesion molecule antisense (lncRNA DSCAM-AS1) is regulated by estrogen and mediates tumor progression in hormone-dependent breast cancer [100]. Following DNA damage, RNA polymerase II recruits the MRE11 (MRE11 homolog, double strand break repair nuclease)-RAD50 (RAD50 double strand break repair protein)-NBS1 (Nijmegen breakage syndrome 1) complex to double-strand break sites and synthesizes damage-inducing lncRNAs [101]. Recently, Kim et al. identified a role of MYC-regulated lncRNAs, named MYCLos, in cell cycle progression and colorectal tumorigenesis through regulating p15 and p21 [102]. Moreover, an antisense transcript of homeobox C10 (lncRNA HOXC-AS3) is obviously activated by increases of H3K4me3 and H3K27ac in gastric cancer, suggesting that lncRNAs can be regulated by histone modifications [103]. 

### 4.2. LncRNA Turnover

LncRNAs can regulate gene expression at the level of transcription by interference with mRNA expression, competition at genomic loci for transcription factors, or chromatin remodeling. Furthermore, lncRNAs influence pre-mRNA splicing, nuclear trafficking, and mRNA/protein degradation at the post-transcriptional level. Based on the emerging emphasis of lncRNAs on regulating gene profiles, the metabolism of lncRNA itself, termed lncRNA turnover, will likely be a vital aspect of its function. Several studies have provided new insights into lncRNA turnover, as follows. Decapping regulates the level or clearance of functional lncRNAs in different contexts because lncRNAs will be accumulated when decapping mRNA 2 (DCP2)-dependent decapping is blocked [104,105]. However, whether the regulation of lncRNA turnover by DCP2-dependent decapping contributes to cancer onset and progression requires further investigation. Moreover, lncRNA expression has been found to be more affected than mRNA expression by a deficiency in the poly(A)-binding protein nuclear 1 (PABPN1), which effectively promotes lncRNA degradation via a polyadenylation-dependent mechanism [106].

### 4.3. Regulation of LncRNAs by Epitranscriptomics

Both the structure and function of lncRNA will likely be affected by nucleoside/RNA modifications. 5-Methylcytidine (m^5^C), N6-methyladenosine (m^6^A), and adenosine to inosine (A-to-I) editing have been documented in lncRNA modification [107]. LncRNAs XIST and HOTAIR harbor m^5^C modification sites in functionally relevant regions, which modulate their interactions with chromatin-modifying complexes [108]. One of the most abundant and dynamic mRNA modifications is m^6^A, in which a methyl group is attached to the N6 position of adenine [109,110]. Methylated RNA immunoprecipitation sequencing (MeRIP-seq) has been identified in more than 1500 m^6^A modification sites in lncRNAs, accounting for ~12% of the total m^6^A peaks [111,112]. Hairpin-stem structures of lncRNA MALAT1 contain two m^6^A residues, altering the secondary structure of MALAT1 to facilitate the binding of heterogeneous nuclear ribonucleoprotein C (hnRNPC) responsible for pre-mRNA processing [113,114]. The m^6^A reader YTH N6-methyladenosine RNA binding protein 3 (YTHDF3) binds m^6^A-modified lncRNA GAS5 to promote its decay [71]. Interestingly, m^6^A methyltransferase-like 3 (METTL3) depletion reduces the binding of DGCR8 to primary miRNA (pri-miRNAs) and leads to an accumulation of unprocessed pri-miRNAs [115,116], indicating that m^6^A can mark pri-miRNAs for processing. LncRNA transcripts can serve as pri-miRNA precursors; however, it is not clear whether m^6^A can regulate lncRNA processing. Hydrolytic deamination of adenosines (A) to inosines (I) is catalyzed by the adenosine deaminases acting on RNA (ADARs). Although A-to-I editing sites have been identified by RNA-Seq analysis in many lncRNAs, including MALAT1, XIST, and 7SK [117,118,119], the effects of A-to-I editing on particular lncRNA are not fully understood. A recent study suggests that prostate cancer antigen 3 (lncRNA PCA3) forms double-strand RNA with prune homolog 2 with the BCH domain (PRUNE2), which undergoes ADAR-dependent A-to-I RNA editing to regulate PRUNE2 levels [120].

## 5. Diverse Mechanisms of LncRNAs: An Example from Autophagy in Cancer

Autophagy is a highly regulated cellular degradation system, involving the sequestration of cytoplasmic components within the autophagosome, which is a transient double-membrane organelle. The autophagosome then fuses with the lysosome, resulting in the degradation of autophagic cargo to maintain cellular homeostasis [121,122]. The dysregulation of autophagy is involved in many human diseases, including cancers. Autophagy acts as a tumor suppressor by degrading the damaged proteins or organelles; however, autophagy sometimes promotes tumorigenesis exhibiting a survival/escape mechanism [123,124]. Here, we summarize the molecular mechanisms underlying lncRNA participants in autophagy regulatory networks, mainly through the regulating core machinery of autophagy (e.g., autophagy-related proteins and LC3) or autophagy-related proteins (e.g., mTOR, ULK1, and SIRT1) (Table 5).

LncRNA MALAT1 attenuates the inhibitory effect of miR-23b-3p on target autophagy-related 12 (ATG12), leading to chemo-induced autophagy and chemoresistance in gastric cancer [125]. In pancreatic ductal adenocarcinoma, lncRNA PVT1 acts as a sponge to regulate miR-20a-5p and thus affects the expression of unc-51-like-kinase 1 (ULK1), which is a key molecule of autophagy initiation [126]. Human maternally expressed gene 3 (lncRNA MEG3) promotes autophagy by protecting autophagy-related 3 (ATG3) from degradation in ovarian cancer [127]. Highly upregulated in liver cancer (lncRNA HULC) upregulates deubiquitinase ubiquitin specific peptidase 22 (USP22), protecting the ubiquitin-mediated degradation of a histone deacetylase Sirtuin 1 (SIRT1) protein to further trigger autophagy [128]. The antisense transcript of nicotinamide phosphoribosyltransferase (lncRNA NAMPT-AS) recruits POU class 2 homeobox 2 (POU2F2) to activate the transcription of *NAMPT*, in order to further promote tumor progression and autophagy in triple-negative breast cancer through the mTOR pathway [129]. LINC00470 regulates the methylation level of extracellular leucine-rich repeat and fibronectin type III domain containing 2 (ELFN2) by decreasing H3K27me3 occupancy in glioblastoma cell autophagy [130]. LncRNA MALAT1 mediates autophagic activation through regulating HuR-TIA1 (TIA1 cytotoxic granule-associated RNA binding protein), which is a major regulator of mRNA stability and translation [131]. The eosinophil granule ontogeny transcript (lncRNA EGOT) recruits heterogeneous nuclear ribonucleoprotein H1 (hnRNPH1) to enhance the alternative splicing of pre-inositol 1,4,5-trisphosphate receptor type 1 (ITPR1) to promote autophagy in human cancer [132].

## 6. Conclusions

Studies conducted over the past 10 years have indicated that lncRNAs are implicated in a range of developmental processes and diseases, including cancer. In the current article, the diverse molecular mechanisms of lncRNAs during tumorigenesis have been comprehensively summarized, including competing ceRNA mechanisms, epigenetic regulation, decoy and scaffold mechanisms, mRNA and protein stability regulation, transcriptional and translational regulation, miRNA processing regulation, the architectural role of lncRNAs, and other uncommon mechanisms. Detailed elucidation of the role of lncRNAs and their regulatory mechanism will help to comprehensively understand the central dogma of DNA-RNA-protein, the evolution of complexity in higher eukaryotes, and the progression of cancers. Furthermore, determining the structure of lncRNAs and the complex of lncRNA is of significant importance for exploring the detailed mechanism of action of lncRNAs. Moreover, lncRNA itself is dynamically regulated in cancer cells, involving the regulation of its expression, turnover (metabolism), and modifications.

It will also be important to explore whether lncRNAs could be used in the diagnosis, prognosis, and therapeutics of cancer. Few lncRNAs are already implicated as biomarkers of cancer diagnosis and prognosis indexes in clinical trials (Table 6); however, there are still many challenges and validation is required for their clinical application. LncRNA-based cancer therapies are promising; however, at present, screening small-molecule libraries to identify candidate lncRNAs and the development of delivery strategies are required prior to clinical trials. A recent study functionalized lncRNAs in drug resistance by integrated genome-wide CRISPR activation of the lncRNA strategy, targeting 14,701 lncRNA genes, and found that growth arrest specific 6 antisense (lncRNA GAS6-AS2) triggers hyperactivation of the GAS6/TAM (TYRO3-AXL-MER2K) pathway, which is a known drug resistance mechanism in multiple cancers [133]. Novel identified lncRNAs, novel lncRNA modifications and turnover, and the novel functions and mechanisms of lncRNAs will be reported in the near future to further extend our understanding of the non-coding RNA world. An in-depth understanding of lncRNAs will not only shed light on their functions in cancer biology, but will open novel avenues for cancer diagnosis and therapeutics.

## Figures and Tables

**Table 1:** Examples of the competing endogenous RNA (ceRNA) mechanism.

LncRNA	miRNA	mRNA	Cancer Type	Ref
PTENP1	*PTEN*-targeting miRNAs	*PTEN*	Various tumor cells	[27]
LINC00673	miR-150-5p	*ZEB1*	Lung cancer	[28]
ODRUL	miR-3182	*MMP2*	Osteosarcoma	[29]
LINC01234	miR-204-5p	*CBFB*	Gastric cancer	[30,31]
NEAT1	miR-34a	*SIRT1*	Colorectal cancer	[32]
WDFY3-AS2	miR-18a	*RORA*	Ovarian cancer	[33]

**Table 2:** Epigenetic regulation by lncRNAs.

LncRNA	Chromatin Remodeling Complexes	Locus	Cancer Type	Ref
ANRIL	PRC1 (CBX7)	*INK4b-ARF-INK4a*	Prostate cancer	[45]
HOTAIR	PRC2	*HOXD cluster*	Breast cancer	[46]
PTV1	PRC2 (EZH2)	*p15, p16*	Gastric cancer	[47]
LINK-PINT	PRC2	*Pro-invasive genes*	Lung/colon cancer	[48]
FGFR2-AS	PRC2/KDM2a	*FGFR2*	Epithelial cell	[49,50]
HOXD-AS1	WDR5	*Target genes*	Prostate cancer	[51]
GCAWKR	WDR5/KAT2A	*Target genes*	Gastric cancer	[52]
GAS8-AS1	MLL1/WDR5	*GAS8*	Liver cancer	[53]
LncFZD6	SWI/SNF (BRG1)	*FZD6*	Liver cancer	[54]
SATB2-AS1	p300	*SATB2*	Colorectal cancer	[55]
HAND2-AS1	INO80	*BMPR1A*	Liver cancer	[56]
LncHOXA10	NURF	*HOXA10*	Liver cancer	[57]
PYCARD-AS1	DNMT1/G9a	*PYCARD*	Breast cancer	[58]

**Table 3:** LncRNA control of mRNA and protein stability.

LncRNA	LncRNA Interactor	mRNA/Protein Stability	Cancer Type	Ref
UCA1	hnRNPA1	*CDKN2A-p16*	-	[66]
PDCD4-AS1	HuR	*PDCD4*	Breast cancer	[67]
PVT1	-	MYC	8q24-amplified cancer cells	[68]
LINK-A	BRK, LRRK2	HIF1α	Breast cancer	[69]
ANCR	-	EZH2	Breast cancer	[70]
GAS5	-	YAP	Colorectal cancer	[71]

**Table 4:** Identification of lncRNA regulators.

Regulator	LncRNA	Regulatory Mechanism	Cancer Type	Ref
EGF	LIMT	Histone deacetylation of promoter	Breast cancer	[96]
Growth factors	Quiescence-induced lncRNAs	-	-	[97]
Androgen	CTBP1-AS	AR-binding sites	Prostate cancer	[98]
Estrogen receptor β	HOTAIR	Estrogen receptor β binding	Renal cell carcinoma	[99]
Oestrogen	DSCAM-AS1	Oestrogen receptor binding	Breast cancer	[100]
DNA damage stress	Damage-inducing lncRNAs	MRE11-RAD50-NBS1 complex recruitment	-	[101]
MYC	MYCLos	Transcription regulation	Colorectal cancer	[102]
Histone modification	HOXC-AS3	Promotor modification	Gastric cancer	[103]

**Table 5:** LncRNAs regulate ‘autophagy in cancer’ through diverse mechanisms.

LncRNA	Target	Mechanism	Cancer Type	Ref
MALAT1	*ATG12*, miR-23b-3p	ceRNA	Gastric cancer	[125]
PVT1	*ULK1*, miR-20a-5p	ceRNA	Pancreatic cancer	[126]
MEG3	ATG3	Protein degradation	Ovarian cancer	[127]
HULC	SIRT1	Protein degradation	Liver cancer	[128]
NAMPT-AS	POU2F2, mTOR	Scaffold of transcription factor	Breast cancer	[129]
LINC00470	AurkA, eIF2α	Epigenetic regulation	Glioblastoma	[130]
MALAT1	HuR, TIA-1	mRNA stability	Pancreatic cancer	[131]
EGOT	ITPR1	Alternative splicing	Breast cancer	[132]

**Table 6:** Application of LncRNAs in clinical trials.

Identifier	Purpose	Application	Cancer Type
NCT03830619	Evaluate the sensitivity/specificity of serum exosome lncRNA as a diagnosis biomarker	Diagnosis	Lung cancer
NCT04269746	Evaluate the diagnostic value of lncRNA CCAT1 expression	Diagnosis	Colorectal cancer
NCT03469544	Evaluate the value of lncRNA HOTAIR and midkine as biomarkers	Diagnosis	Thyroid cancer
NCT03738319	Screen the candidate lncRNAs as biomarkers for the prognosis	Diagnosis	Epithelia Ovarian Cancer
NCT03057171	Evaluate the expression of lncRNA THRIL and PACER by helicobacter pylori infection	Diagnosis	Stomach cancer
NCT03000764	Seek a molecular signature (e.g., lncRNA) of pathological radiation-induced fibrosis	Diagnosis	Breast cancer
NCT04288739	Detect the prognostic role of lncRNA XIST in acute myeloid leukemia	Diagnosis	Hematologic cancer
NCT03742869	Detect the expression of lncRNAs in uterine cervical adenocarcinoma patients with and without HPV integration	Diagnosis	Uterine cervical adenocarcinoma
NCT04269746	Validate the mRNA-lncRNA signature to predict the efficacy/recurrence risk after a combination of chemotherapy drugs	Prognosis	Triple-negative breast cancer
NCT03742856	Predict the invasiveness and tumorigenesis of cancer cells with different FIGO stages and subtypes	Prognosis	Epithelial ovarian cancer
NCT04010487	Predict the pathogenesis of the malignant transformation of adenomyosis	Prognosis	Endometrial cancer
